# Current Perspective on In Vivo Molecular Imaging of Immune Cells

**DOI:** 10.3390/molecules22060881

**Published:** 2017-05-26

**Authors:** Anushree Seth, Hye Sun Park, Kwan Soo Hong

**Affiliations:** 1 Bioimaging Research Team, Korea Basic Science Institute, Cheongju 28119, Korea; anushree@kbsi.re.kr (A.S.); hspark@kbsi.re.kr (H.S.P.); 2 Immunotherapy Convergence Research Center, Korea Research Institute of Bioscience and Biotechnology, Daejeon 34141, Korea; 3 Graduate School of Analytical Science and Technology, Chungnam National University, Daejeon 34134, Korea

**Keywords:** cellular imaging, image-tracking, immune cells, magnetic resonance imaging, optical imaging

## Abstract

Contemporaneous development of improved immune cell-based therapies, and powerful imaging tools, has prompted growth in technologies for immune cell tracking in vivo. Over the past couple of decades, imaging tools such as magnetic resonance imaging (MRI) and optical imaging have successfully monitored the trafficking patterns of therapeutic immune cells and assisted the evaluation of the success or failure of immunotherapy. Recent advancements in imaging technology have made imaging an indispensable module of immune cell-based therapies. In this review, emerging applications of non-radiation imaging modalities for the tracking of a range of immune cells are discussed. Applications of MRI, NIR, and other imaging tools have demonstrated the potential of non-invasively surveying the fate of both phagocytic and non-phagocytic immune cells in vivo.

## 1. Introduction

Immune cells contribute to the pathogenesis or treatment of a wide variety of diseases, such as autoimmune and infectious diseases, and immunotherapy-based treatments. Components of innate immune systems include monocytes, dendritic cells (DCs), macrophages, granulocytes, natural killer (NK) cells, and other cells. They represent the first line of defense and are less specific than the cells of the adaptive immune system, predominantly comprising T cells and B cells. Each cell type has a well-defined role in the cascade of events that occurs following encounter with a pathogen or transformed cells. For example, (1) macrophages get rid of cell debris and pathogens; (2) DCs and macrophages act as major antigen-presenting cells and direct the inflammatory response by secreting cytokines, and other factors; and (3) T cells and NK cells directly kill virus-infected cells and cancer cells [1]. Apart from these beneficial effects of immune cells, these cells are also known to have unfavorable effects on the human body in certain conditions. For instance, immune cells might become responsive to self-antigens, thereby causing auto-immune diseases such as rheumatoid arthritis, diabetes mellitus type 1, inflammatory bowel disease, Graves’ disease, multiple sclerosis, and systemic lupus erythematosus [2]. Furthermore, immune cells such as macrophages and regulatory T cells are known to infiltrate tumor and facilitate tumor growth [3,4,5]. Immunosuppression is also required during allograft transplantation for preventing graft vs. host reactions. As immune cells are indispensable components of immunotherapeutic interventions and various pathophysiological conditions, tracking their presence inside the body can provide vital information. For decades, researchers have been looking for novel approaches to track immune cells in their native environment. With the development of immune cell-based therapies, it has become increasingly important to visualize the bio-distribution of the injected cells. The success or failure of immune cell-based therapy can primarily be assessed by tracking the presence of cells at the site of interest.

**Figure 1:**
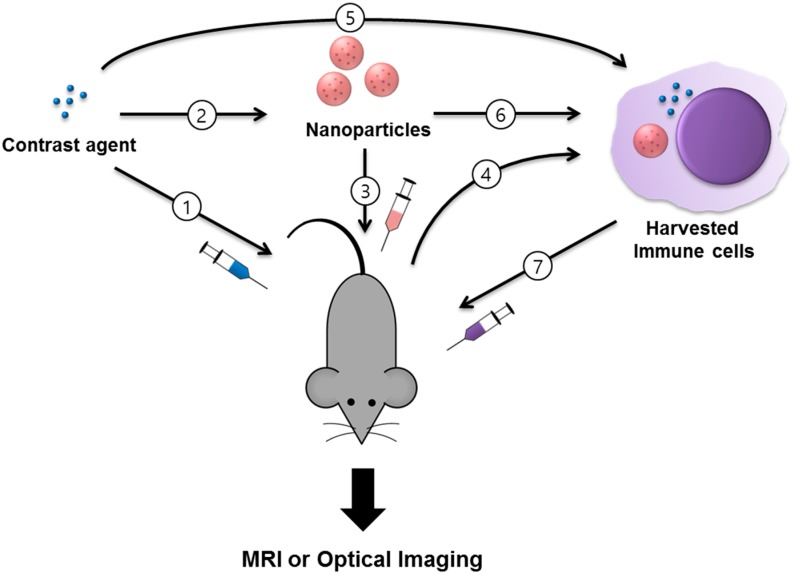
Schematic representation of strategies for immune cell tracking by magnetic resonance imaging (MRI) or optical imaging. (1) A T_1_ or T_2_ contrast agent is directly injected or (2) formulated as or encapsulated in nanomaterials for (3) in situ labeling of immune cells and imaging by MRI or optical imaging; (4) Immune cells can also be harvested and labeled ex vivo with (5) a contrast agent or (6) nanoparticles before (7) injection and MRI-based tracking, distribution, and behavior of immune cells in vivo.

**Figure 2:**
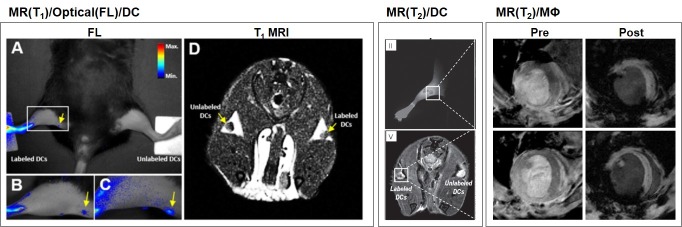
In vivo immune cell tracking by magnetic resonance and optical imaging modalities, for DCs migrating in lymph node (**left** and **middle**), and macrophages (MΦ) infiltrated in myocardial infarction (**right**). Adapted with permission from Kim et al. [11] (Copyright 2016 American Chemical Society) and Kim et al. [12].

Non-invasive cell tracking can broadly be classified into two types: direct labeling of cells with a contrast agent (CA) that is imaged using appropriate instruments; or indirect labeling of cells, which includes reporter genes [6,7,8]. Direct labeling of immune cells with CAs can be either in vivo or ex vivo as depicted in Figure 1. The various non-radiation based imaging tools being used for immune cell tracking include magnetic resonance imaging (MRI), optical imaging, and bioluminescence imaging [9,10] (Figure 2). While each of these methods have their own limitations and advantages, biocompatibility and non-toxicity of CAs are essential for clinical translation of these imaging approaches. Furthermore, researchers are aiming to improve the specificity of CAs for better image resolution.

## 2. Imaging Modalities

### 2.1. MRI (T_1_, T_2_)

The human body predominantly consists of water. In the presence of an external magnetic field, hydrogen nuclei (^1^H or proton) in the water molecules align with the magnetic field. Signals from fluorinated molecules (^19^F) can also be used for MRI. ^1^H or ^19^F nuclei are disturbed from this equilibrium by pulsed radio frequency radiation. After the removal of the radio frequency radiation, nuclei reach the equilibrium and induce a short-lived voltage in a receiver antenna; this transient voltage is the nuclear magnetic resonance (NMR) signal. MRI is a powerful technique for non-invasive molecular imaging that provides soft tissue contrast with high anatomical resolution without the use of potential toxic radiation [11]. T_1_ or T_2_ is the time that it takes for the longitudinal or transverse magnetization to decay to 63% or 37% of its original value. These values are characteristics of specific tissues, depending on the main magnetic field strength in a measuring system. Contrast-enhanced MRI using positive (T_1_) or negative (T_2_) CAs supports the diagnosis of diseases and follow-up evaluations of treatments with detailed tissue contrast. MR CA-based delivery systems for the diagnosis and therapy of cancers have been widely studied, and various CA modifications have been developed for specific cancers [13,14].

With recent developments in the field of cancer immunotherapy, there is increasing interest in MRI-based in vivo cell tracking techniques [10,15,16]. Magnetic nanoparticle (MNP)-labeled immune cells have been used to track cell behaviors in deep tissue areas in vivo to elucidate cellular immunological processes within diseased tissue environments and draining lymph nodes [17,18]. Wu et al. have monitored migration of MNP-labeled DCs into lymph nodes by in vivo T_2_-weighted MRI [18]. MNP labeling of phagocytic immune cells has been successfully performed by controlling their surface characteristics or the addition of transfection agents [6,10,19,20]. Efficient tracking of labeled cells, enabling long-term, sensitive detection in vivo, depends on the magnetic properties of MNPs. Superparamagnetic iron oxide (SPIO) nanoparticles as T_2_ CAs have been extensively studied in labeling and image-tracking of immune cells, and are a useful tool for medical applications [9,21,22]. Mou et al. have reported SPIO-enhanced green fluorescent protein-labeled DCs homing to the draining lymph nodes by in vivo MRI [22]. Moreover, gadolinium chelating T_1_ CA demonstrates intense positive signals over background tissues [11]. Meanwhile, non-phagocytic cells such as NK cells are difficult to label with particulate CAs, and therefore require additional treatments such as labeling with cationic materials or electrostatic forces in cellular environments [23,24].

### 2.2. Optical Imaging

Optical imaging (OI) methods have been widely used for visualizing cells and soft tissues in various biomedical fields. Using visible light (400–700 nm), OI easily distinguishes specific cells or intracellular living organisms simultaneously, along with the selectivity of imaging colors. OI methods include fluorescence and bioluminescence imaging (BLI), which are non-invasive and has reduced damaging effects from radiations by using non-ionization radiation. It enables long-term tracking of cells with sensitive detections, but its limitations are a low penetration depth of only 1–2 mm, and low spatial resolution by scattered lights generated within tissues. In this regard, use of near-infrared (NIR) region (700–1000 nm) in OI is preferred to increase detection sensitivity [25,26,27].

Organic fluorophores for fluorescence imaging are generally used for staining of nuclei, lysosomes, cell membrane, and various proteins in cellular organisms. For the tracking of immune cells, especially non-phagocytic cells, lipophilic fluorescent dyes have been widely used due to their efficient staining process and tolerable cytotoxic levels [28]. Tavri et al. have reported image-tracking of lipophilic dye, DiD(1,1′-dioctadecyl-3,3,3′,3′-tetramethylindodicarbocyanine)-labeled, tumor-targeted NK cells to human prostate cancer xenografts with in vivo fluorescence imaging [28]. However, their usefulness in supplying quantitative information for the tracking cells is limited owing to photo-bleaching or biodegradation after in vivo administration.

BLI is an indirect cell labeling with reporter genes, based on the detection of the light generated from various luciferase enzymes, such as firefly luciferase (FLuc), *Renilla* luciferase (Rluc) or bacterial luciferase [29,30]. BLI of immune cells was successfully performed by labeling cells with fluorescent proteins or luciferase reporter genes by transfection, which supports the photo stability of fluorescent signals compared to organic fluorophores [9,30]. BLI can provide images of higher sensitivity compared to fluorescence imaging, by detection of emitting light from specific substrates without the auto fluorescence generated by excitation light.

Another emerging technique for OI is Cerenkov luminescence imaging (CLI), which is based on the detection of visible photons emitted by Cerenkov radiation and provides great potential for rapid application into clinical practice [31].

Image-tracking of immune cells by OI techniques enables in vivo real-time monitoring of therapeutic effects for immune cell-based therapy. For clinical applications, in vivo OI methods could allow easy optimizations of therapeutic immune cells from several candidates of specifically engineered cells.

### 2.3. Miscellaneous: Upconversion Nanoparticles and Quantum Dots

As an alternative to OI materials, nanocrystal-structured nanomaterials such as quantum dots (QDs) have been developed to improve photo-stability during cell tracking; they possess unique luminescent characteristics and electronic energy properties such as broad absorption spectrum and narrow emission levels, showing high photostability for various biological applications. However, their high cytotoxicity remained a concern for in vivo applications [32]. Recently, upconversion nanoparticles (UCNPs) have attracted increasing attention in the field of immune cell labeling and tracking because of their high resistance to photobleaching and quantitative sensitive detections [33,34]. UCNPs enable NIR-to-NIR imaging by absorbing NIR excitation light and emitting NIR luminescence by an upconversion energy transfer process, which improves signal-to-noise ratio with absence of auto fluorescence [33,35,36]. UCNPs are also useful for multicolor imaging by controlling dopant ions and multimodal imaging with MR, single-photon emission computed tomography (SPECT), or computed tomography (CT) imaging [37,38]. Recent studies on immune cell tracking, depending on imaging modalities, cell types, applications, and CAs, are summarized in Table 1.

## 3. Applications of Imaging Immune Cells

### 3.1. Macrophages/Monocytes

#### 3.1.1. Tumor-Associated Macrophages and Immunotherapy

Tumor microenvironments are heterogeneous with a variety of infiltrated cells including macrophages. Macrophages that reside inside or in close proximity to tumors and assist tumor progression are primarily classified as tumor-associated macrophages (TAMs). They facilitate formation of tumors by secreting pro-angiogenic factors and tumor immune evasion, promoting metastasis and neoplastic transformation. TAMs are present in various types of human tumors such as glioblastoma, and lymphoma, breast, prostate, thyroid, and ovarian tumors leading to rapid cancer progression and a decrease in patient survival [5,42]. Labeling TAMs with an appropriate CA for imaging in a non-invasive manner can also help in guiding biopsies. The number of TAMs present in tumor tissue also helps in envisaging the efficiency of therapeutic intervention.

Ultra-small superparamagnetic iron oxide nanoparticles (USPIONs) were used for MRI of TAMs in breast cancer [43]. After intravenous injection of the Food and Drug Administration (FDA) approved iron oxide nanoparticle compound ferumoxytol (Feraheme™) it was found to be preferentially taken up by TAMs rather than by breast cancer cells, thus making it a clinically applicable approach. In another study, USPIO were compared with per-fluorocarbon (PFC) agents for imaging TAMs present in breast cancer [44]. While imaging post-USPIO suffered signal loss, ^19^F imaging provided better information about spatial distribution and density of TAMs.

Magneto-fluorescent particles (dextran-coated iron oxide core nanoparticles coupled with fluorochrome VT680) were used for non-invasive tracking of TAMs, at different resolutions and using various imaging modalities, e.g., fluorescence molecular tomography (FMT), MRI, and multiphoton and confocal intravital microscopy [45]. Both mesoscopic and macroscopic imaging modalities, e.g., FMT, MRI, and FMT-MRI fusion imaging, were tested for studying the in vivo distribution of AMTA680-labeled TAMs at the whole tumor (or body) level. Using FMT reconstruction and quantification of three-dimensional maps of AMTA680 was possible along with visualization of well-delimited signal foci within the tumor. MRI allowed identification of foci of hyposignal (black) on T_2_-weighted images at submillimeter levels. Signal co-localization was illustrated after fusion of FMT and MRI data sets. These nanoparticles enabled the estimation of cellular activity and biodistribution of TAMs.

#### 3.1.2. Cardiovascular Diseases: Myocarditis, Myocardial Infarction, and Aneurysm

Cardiovascular magnetic resonance (CMR) imaging with magneto-fluorescent nanoparticles exemplified a better evaluation of level of inflammation in rats with experimental autoimmune myocarditis (EAM) as compared to conventional CMR [17]. MNP uptake by infiltrating inflammatory cells lead to altered myocardial T_2_ effect.

The heart undergoes a wound healing process after ischemia or myocardial infarction, which is initiated by cells of the innate immune system. These cells mainly comprise macrophages that are recruited at the site of injury for phagocytosis of necrotic tissues. Visualization of these recruited macrophages provides important diagnostic information for preventing irreparable heart failure. Gadolinium-loaded liposomes were used as T_1_ shortening CA for labeling and imaging of monocytes and/or macrophages [46]. Images of the labeled mice, provided information on the spatiotemporal distribution of macrophages post-myocardial infarction, compared to that of unlabeled mice. MRI for monitoring of cardiovascular pathology has also been tested in humans. A clinical trial investigated the potential of CMR imaging using ferumoxytol, a USPION, for comprehensive characterization of infarct pathology and compared it with gadolinium-based imaging in patients with acute myocardial infarction [47]. It was found that in humans, USPIO-based CAs detected infiltrated macrophages in myocardial infarct, which subsequently provided a more detailed characterization of myocardial infarct pathology.

#### 3.1.3. Inflammation and Ischemia

**Figure 3:**
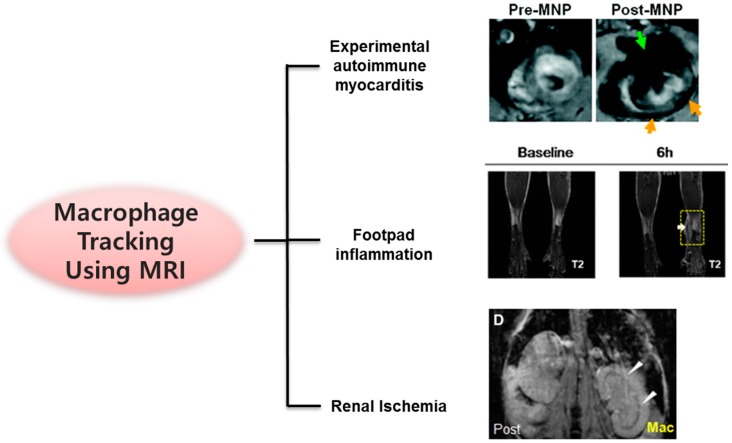
Schematic representation of various applications of magnetic resonance imaging (MRI) for tracking of macrophages. Adapted from Moon et al. [17], Cai et al. [48], and Kang et al. [49] with permission of Springer. MNP: magnetic nanoparticle.

SPIONs were used for labeling and tracking of intravenously administered macrophages in renal ischemia-reperfusion mouse models [48]. MRI of macrophages homing to a damaged kidney may enable timely investigation of the pathogenesis of acute kidney injury and provide cues for determining a treatment for acute renal failure. In another study, MRI was combined with NIR fluorescence imaging for tracking of macrophage migration to the site of inflammation [49]. The multimodal imaging nanoparticles offered non-invasive imaging in a living organism, owing to their biocompatibility due to the presence of silica-coated MNPs encapsulating NIR fluorescence dye within the silica shell and magnetic core. The migration of primary macrophages in living mice with acute inflammation induced by an injection of carrageen solution into the footpad was successfully tracked using both MRI and NIR imaging. Moreover, the impact of dexamethasone, a potent steroid hormone with anti-inflammatory effects, on the migration of macrophages to inflammatory lesions was effectively visualized. Several cases on in vivo MR imaging of macrophages are shown in Figure 3.

### 3.2. T Cells

Antigen-specific T-lymphocytes have received considerable attention as a novel modality for cancer therapy [50,51]; however, to better understand the in vivo fate of therapeutic cells including their bio distribution, homing and migration to the target site, there is a need to track these cells non-invasively. A major impediment for a detailed estimation of antitumor efficacy has been the inability to track T lymphocytes in vivo at desirable spatio-temporal resolutions. The existing strategies involve bulk distribution study of radiolabeled cells or bioluminescence imaging of luciferase-transfected cells [52].

A biocompatible, dextran coated SPIO particle was derivatized with a peptide sequence from the HIV-tat protein to improve intracellular magnetic labeling of T lymphocytes [39]. It was found that these nanoparticles exposed the three-dimensional spatial heterogeneity of T-cell recruitment to tumors and verified the temporal regulation of T-cell recruitment within the tumor. The recruitment of cytotoxic T lymphocytes to tumors over longer times was studied using MRI and it revealed a time-dependent heterogeneity and detection limits. A signal reduction was observed at 48 h after administration, but the same animal showed no signal reduction by MRI and returned to baseline signal intensity at 60 h after adoptive transfer. Distribution of activated PFC-labeled ovalbumin-specific T cells from the T cell receptor-transgenic line OT-1 was determined in vivo by ^19^F-MRI/MRS (magnetic resonance spectroscopy) [53].

### 3.3. Dendritic Cells

Immunotherapeutic DCs are being engineered to stimulate helper or killer T cells in vivo. Monitoring in vivo behavior and interaction of DCs is crucial, and various imaging modalities have been reported, including MRI, reporter gene based imaging, gamma scintigraphy with In-111, fluorescence imaging, bioluminescence imaging, QD and positron emission tomography (PET) with ^18^F. Immature DCs are known to regularly undergo endocytosis for sampling their surrounding environment for potential foreign entities. After encountering any form of danger signal they are expected to undergo activation and maturation, which induces downregulation of their endocytic machinery. Therefore, for efficient labeling of DCs with adequate quantity of CAs, immature DCs are superior to mature ones. Not only the maturation status, but also the timeline (sequential vs. simultaneous) of antigen and contrast agent delivery must be taken in to consideration. Simultaneous delivery of antigen and CA might interfere with antigen processing leading to inappropriate presentation on MHC I (major histocompatibility complex I) and MHC II.

A dual imaging probe including gadolinium and the NIR fluorophore, aza-boron-dipyrromethene, was used for tracking DCs in lymph nodes and subsequently could be used in the investigation of advanced immunotherapy [11]. In another study, electrostatically assembled and crosslinked, biocompatible polyelectrolyte-coated MNPs were used for bimodal imaging of DCs in lymph nodes [12]. Bimodal imaging is advantageous as it exploits the complementary strengths of each modality. In a report, iron oxide nanoparticles and indocyanine green in a poly(lactide-*co*-glycolide) matrix were used for studying migration of DCs via lymphatic drainage in real time [54]. Not only is the mapping of lymph nodes crucial, but the clearance and bio-distribution of CA is also important. Recently, UCNPs were used for development of high-resolution images of mapping and clearance of UCNPs [55].

Interestingly, an MRI reporter gene, human ferritin heavy chain (FTH), was introduced in the DC2.4 cell line using a lentivirus, and was found to be a useful technique for longitudinal monitoring of DCs and estimating their therapeutic efficacy [7]. FTH-DC did not influence the migration, proliferation, and co-stimulatory marker upregulation capacity, which is critical for development of effective DC-based vaccines.

### 3.4. Natural Killer Cells

Autologous and NK cell line-based immunotherapeutic approaches have drawn significant attention as a powerful strategy for treatment and management of cancer. However, there are only limited reports on in vivo imaging of NK cells, primarily because of difficulties in labeling NK cells with imaging probes. Genetically modified therapeutic NK cells that are capable of targeting tumor-associated antigens need to be monitored in real time in vivo in a non-invasive manner for determining their tumor-homing capacity.

The nonradioactive isotope ^19^F was optimized in a dose-dependent manner for non-invasively labeling and tracking NK cells [56]. ^19^F was passively taken up by NK cells, and without affecting viability, cytotoxicity, cytokine secretion and activation. In another report, KHYG-1 NK cells were detected by MRI after subcutaneous injection, but not intravenous or intraperitoneal injection [39]. NK cells are non-phagocytic cells that are difficult to label by simple incubation. Both NK-92 and NK-92-scFv(FRP5)-zeta cells were successfully labeled with ferucarbotran and ferumoxides by lipofection and electroporation, but not by simple incubation [57]. Furthermore, accumulation of these labeled cells with clinically applicable T_2_ CAs in murine tumors can be monitored in vivo with MRI. In a similar study, NK-92-scFv(MOC31)-zeta cells, which express a chimeric antigen receptor (CAR) specific for the tumor-associated EpCAM (epithelial cell adhesion molecule) antigen, were labeled with SPIO ferumoxides and exhibited a progressive and a significant drop in contrast-to-noise-ratio data at 1 h and 24 h post-injection [23]. SPIONs were used for quantitative visualization of transcatheter intra-arterial delivered NK cells to hepatocellular carcinoma, and ΔT_2_* was found to be higher in the tumors than in the normal liver tissue (*p* < 0.001) [58].

Human NK-92MI cells were labeled with an anti-CD56 antibody conjugated with QDs (QD705) without compromising their viability, IFN-γ production, and cytolytic activity. In human malignant melanoma (MeWo) xenografts in mice, the labeled NK cells could be tracked for up to 12 days following intratumoral injection [41]. In another study, fluorophore DiD (1,1’-dioctadecyl-3,3,3’,3’-tetramethylindodicarbocyanine)-labeled NK-92-scFv(MOC31)-zeta cells targeting the EpCAM antigen on prostate cancer cells exhibited a substantial increase in tumor fluorescence at 24 h post-injection [28]. Cy5.5-conjugated magnetic iron oxide (Fe_3_O_4_) nanoparticles controlled the movement of human NK (NK-92MI) cells in vivo under the effect of an external magnetic field and enabled in vivo monitoring using in vivo imaging system [59].

## 4. Limitations of Existing Cell Tracking Approaches and Future Prospects

For clinical application, an imaging method should be able to evaluate both cellular delivery and therapeutic effectiveness in patients. Moreover, it must be non-invasive and nontoxic, and permit a precise and quantitative assessment of the cell-based therapy. Owing to different membrane properties and differential ability to phagocytose, direct labeling of immune cells ex vivo is a challenging task. In addition, the ability to retain the CA in vivo is advantageous for terminally differentiated cells; otherwise, the signal may be diluted or lost because of cell proliferation or death. ^19^F MRI has a detection limit in vivo of approximately 10^4^ cells per cm^3^, which prevents detection of cells after migration to the tumors post-intravenous or percutaneous injections. Moreover, in cases of labeled cell death, phagocytic cells such as macrophages and DC could take up cell debris and lead to false positive signals. Immune cell therapies with T cells or NK cells in cancer have emerged as promising strategies. One approach is to express CAR on the T cell or NK cell membrane (CAR-T or CAR-NK), which has been widely used to confer a desired specificity as targeted therapy for cancer. However, there are several concerns to address before its clinical applicability such as bio-distribution in the whole body, when and how many transplanted cells infiltrate tumor tissue, cell survival, and anticancer efficacy. To address these issues, multiplexed OI probes can be used for simultaneous in vivo tracking of different cell phenotypes. Following intravenous administration of about 10^6^ cells per mouse, quantitative time/spatial information of the cells using in vivo MRI/OI may provide a systematic selection of parameters for immunotherapy including cell dose, treatment time/interval, choice of CAR, selection of cancer cell type, and combination therapy with chemical drugs or antibody.

## 5. Conclusions

Overall, for future clinical applications, multimodal imaging (MRI, NIR and UCNP) is anticipated to be the most desirable technique for qualitatively and quantitatively tracking immune cells, by visualization of signal co-localization. The in vivo kinetic behavior of immune cells is intricate and poorly understood. Progress in immune cell-based therapies has encouraged the development of imaging tools with single-cell precision and sensitivity, for guiding and conducting dynamic monitoring of experimental therapies. The detailed understanding gained from the improved imaging modalities will help enrich immune cell based therapeutic approaches, which is likely to provide an impetus towards their clinical application. PFC, SPIO, and NIR-based immune cell imaging has already reached clinical trials and further advancements are expected in time to come.

## Figures and Tables

**Table 1:** Recent studies on in vivo immune cell tracking by MR and optical imaging modalities.

Imaging Modality	Type	Labeled Cell Type	Contrast Agent	Animal Model	Applications (Target)	Tracking Time	Administration	Ref.
MR	T_2_	T cell	SPIO	Tumor	B16 melanoma cell	36 h	i.p.	[39]
MR	T_2_	Dendritic cell	SPIO	Immunized	Lymph node mapping	72 h	footpad	[18]
MR	T_2_	NK-92-scFv(MOC31)-zeta cells	SPIO	Tumor	EpCAM-positive DU145 prostate cancer cell	24 h	i.v.	[24]
MR	T_2_	Novel NK cell line (KHYG-1)	USPIO	Tumor	PC-3M human prostate cancer cell	4 days	i.v., i.p., s.c.	[40]
MR/Optical	T_2_/BLI	Macrophage/monocyte	SPIO	Stroke	Brain imaging	72 h	i.v.	[29]
MR/Optical	T_1_/FL	Dendritic cell	Gd	Normal	Lymph node mapping	24 h	footpad	[11]
Optical	FL	NK-92-scFv(MOC31)-zeta cells	DiD	Tumor	EpCAM-positive DU145 prostate cancer cell	24 h	i.v.	[28]
Optical	FL	NK92MI	QD	tumor	MeWo human melanoma cell	24 h	i.t.	[41]
Optical	PL	Mouse mesenchymal stem cell	UCNP	Normal	Biodistribution	24 h	s.c.	[34]
Optical	PL	Dendritic cell	UCNP	Immunized	Lymph node mapping	48 h	footpad	[33]

MR: magnetic resonance; BLI: bioluminescence imaging; FL: fluorescence; PL: photoluminescence; Gd: gadolinium; NK: natural killer; DiD: 1,1′-dioctadecyl-3,3,3′,3′-tetramethylindodicarbocyanine; EpCAM: epithelial cell adhesion molecule; SPIO: superparamagnetic iron oxide; QD: quantum dot; UCNP: upconversion nanoparticle; USPIO: ultra-small superparamagnetic iron oxide; i.v.: intravenous; i.p.: intratumoral; s.c.: subcutaneous.
